# A Mobile Game Intervention for Young Persons Living With HIV and Depression in Nigeria: Protocol for a Pilot Randomized Controlled Trial

**DOI:** 10.2196/74199

**Published:** 2025-12-03

**Authors:** Caleb Eliazer, Temitope Omotosho, Kehinde M Kuti, Leslie J Pierce, Dalton Gray, Carolyn M Audet, Olutosin Awolude, Oye Gureje, Bibilola Oladeji, Aima A Ahonkhai

**Affiliations:** 1 Medical Practice Evaluation Center Massachusetts General Hospital Boston, MA United States; 2 Infectious Disease Institute College of Medicine University of Ibadan Ibadan Nigeria; 3 Staff Medical Services Department University College Hospital Ibadan Nigeria; 4 Overlook Games New York City, NY United States; 5 Vanderbilt University Medical Center Nashville, TN United States; 6 Department of Obstetrics and Gynaecology College of Medicine University of Ibadan Ibadan Nigeria; 7 Department of Psychiatry College of Medicine University of Ibadan Ibadan Nigeria; 8 Division of Infectious Diseases Massachusetts General Hospital Boston, MA United States; 9 Center for AIDS Research Harvard University Boston, MA United States

**Keywords:** HIV comorbidities, young people living with HIV, cognitive behavioral therapy, problem-solving therapy, HIV, mental health, depression, low- and middle-income countries, LMICs, sub-Saharan Africa

## Abstract

**Background:**

Young people living with HIV bear a disproportionate burden of depression, which is associated with poor HIV outcomes. Problem-solving therapy (PST) has been shown to be effective for depression management and can be delivered with fidelity by nonspecialists, especially in resource-limited settings. PST is designed to equip individuals to manage the impact of stressful life events on their mental health. Change My Story is a narrative digital game designed to improve PST engagement among young people living with HIV in Nigeria.

**Objective:**

This trial will evaluate the impact of PST alone or PST with Change My Story on mental health outcomes among young people living with HIV in Nigeria.

**Methods:**

We will conduct a pilot hybrid implementation-effectiveness randomized controlled trial with 80 young people living with HIV (aged 16-24 years) in Nigeria with depression (9-item Patient Health Questionnaire [PHQ-9] ≥9) over 3 months. Participants will be randomized to receive PST with or without Change My Story. All participants will engage in weekly PST sessions for 6 weeks delivered by trained nonspecialists (clinic HIV adherence counselors). At 6 and 10-12 weeks, scores on PHQ-9 will determine the frequency of PST sessions during the remaining intervention period. Primary implementation outcomes, including engagement, satisfaction, feasibility, acceptability, and appropriateness from the participant perspective, will be assessed using validated scales, programmatic data, and focus group discussions at 3 months. Secondary clinical outcomes will assess changes in depressive symptoms, psychological distress, functional disability, antiretroviral therapy adherence, and HIV viral suppression at 3 and 6 months. Implementation outcomes (all but engagement and satisfaction) will be assessed through validated scales and focus group discussions from the implementer perspective at baseline and 6 months.

**Results:**

This study is funded by the US National Institutes of Health (funding commenced on March 8, 2024), institutional review board approval was received on April 15, 2024, and recruitment and data collection began in June 2025. Thus far, we have screened 103 youths and enrolled 23 participants. Among enrolled participants, 15 (65%) were male; 1 (4%) had a PHQ-9 score ≥17, and 6 (26%) had suicidal thoughts. We anticipate recruitment will be completed by January 2026 and follow-up by June 2026. We will assess our hypotheses that PST with Change My Story is feasible, acceptable, and appropriate and that individuals receiving PST integrated with Change My Story will have greater engagement, satisfaction, and depression remission compared to those receiving PST alone.

**Conclusions:**

This pilot randomized controlled trial attempts to establish preliminary data on the feasibility, acceptability, appropriateness, and efficacy of task-shifted PST and supplementary mobile health technology on improving HIV and mental health outcomes among young people living with HIV in Nigeria. These findings may serve as a basis for future large-scale interventions.

**Trial Registration:**

ClinicalTrials.gov NCT06389565; https://clinicaltrials.gov/study/NCT06389565

**International Registered Report Identifier (IRRID):**

PRR1-10.2196/74199

## Introduction

### Background

Approximately 10% of the world’s 4 million young people, aged 15-24 years, living with HIV reside in Nigeria—an African nation with the fourth largest global population of people living with HIV [1-3]. Young people living with HIV also bear a disproportionate burden of depression compared to the general population, which is a key driver of poor HIV outcomes such as higher rates of interruptions in care, medication nonadherence, virologic failure, and progression of HIV [4-7].

Cognitive behavioral therapy (CBT)–based interventions are highly effective in treating depression and have been successfully used across the age spectrum from adolescents to older people [8-10]. Problem-solving therapy (PST) is a CBT-based intervention that is typically administered face-to-face with a trained counselor in 6 to 15 sessions; session duration is typically 30 to 60 minutes. PST uses 7 basic steps to teach problem-solving orientation and skills to equip individuals to manage the impact of stressful life events on their mental health [11,12]. PST interventions are highly effective for the treatment of depression both among adults as well as among women during the perinatal period in Nigeria [13-16]. PST also offers advantages over pharmacologic treatment for mild to moderate depression for people living with HIV, as PST avoids concerns about increasing pill burden and potential drug-drug interactions. Further, PST can be delivered by nonspecialist health workers, which may serve to overcome the critical gap in providing mental health care in Nigeria and other settings with a limited mental health care workforce [17-21].

Completion of PST sessions is required for optimal mental health outcomes; therefore, strategies that maximize adherence to PST may lead to the greatest benefit [11,12,22-24]. Digital games deployed through mobile health (mHealth) technologies present a unique and innovative opportunity to support psychosocial interventions like PST in a youth-friendly manner. Youth-friendly health care is an important care strategy recommended by the World Health Organization (WHO) to address the specialized needs of young people and to promote their engagement in critical health services [23,25-27].

Digital games are played by youths worldwide, and an estimated 71% of adolescents and young adults are connected online [28]. Coupled with the rapid increase in ownership of mobile phones in low- and middle-income countries (LMICs; 90% in Nigeria), digital games provide a compelling format for intervention delivery [29]. A genre of games called serious games is developed specifically for a reason other than pure entertainment (ie, promotion of health behaviors or health outcomes) [30]. One subgenre, narrative digital games, tells stories in an immersive environment, and this appealing format has been leveraged to promote observational learning, cognitive and behavioral rehearsal, and problem-solving for HIV prevention among adolescents and young adults [31,32]. Emerging evidence suggests that interactive games are both feasible and potentially effective tools for supporting chronic disease management and mental health treatment among youths [33-35].

In this paper, we outline a pilot hybrid implementation-effectiveness randomized controlled trial (RCT; ClinicalTrials.gov NCT06389565) to compare Change My Story combined with PST to PST alone among 80 young people living with HIV with depression. We will use the Consolidated Framework for Implementation Research (CFIR) to explore factors influencing engagement, feasibility, acceptability, and satisfaction among young people living with HIV and facilitators and barriers to intervention delivery [36].

### Study Objectives

This 2-arm parallel hybrid implementation-effectiveness study will compare outcomes of PST alone to PST integrated with a novel digital game, Change My Story, for young people living with HIV. Implementation outcomes (primary outcomes) from the perspective of young people living with HIV (feasibility, acceptability, appropriateness, satisfaction, and engagement) will be compared across the 2 study groups. This study will also assess the effectiveness (secondary outcomes) of Change My Story in improving depression, psychological distress, and HIV viral suppression. In addition, we will assess the feasibility, acceptability, and appropriateness of the intervention from the perspective of implementers.

This trial is designed to assess 2 hypotheses. First, we hypothesize that pairing PST with Change My Story will provide a more comfortable entry point into mental health care and thus improve implementation outcomes including engagement, satisfaction, feasibility, acceptability, and appropriateness. It is through this mechanism that we believe PST integrated with Change My Story will have improved preliminary effectiveness on mental health and HIV outcomes compared to PST alone. Second, we hypothesize that integrating PST with Change My Story will be feasible, acceptable, and appropriate from the perspective of implementers.

## Methods

### Study Site

Participants will be recruited in Nigeria from the HIV Clinic, Infectious Disease Institute, College of Medicine, University of Ibadan. The clinic has approximately 6600 patients active in care, with about 400 of these being young people living with HIV. The Infectious Disease Institute also runs a monthly youth clinic, whose activities include a support group and other youth-friendly activities in addition to clinical care for 15- to 24-year-old people. Participants for this study will be recruited from young people living with HIV attending the Infectious Disease Institute by direct outreach to the adolescent clinic with the guidance of our team’s peer champions.

### Intervention Package

We developed Change My Story, a theory-grounded, narrative-based serious game designed to enhance engagement with PST for young people living with HIV in Nigeria. Players of Change My Story navigate emotionally difficult experiences vicariously through the characters and choose a narrative path toward the story’s conclusion. Guided by the social cognitive theory, the game promotes observational learning and self-efficacy by allowing players to explore the consequences of player choice and build problem-solving skills [37,38]. The development process was guided by the Integrate, Design, Assess, and Share framework, a user-centered design approach for digital behavioral health tools [39]. We conducted formative research with young people living with HIV to identify core mental health stressors [40]. This early work highlighted high mobile phone use, frequent gameplay, and a strong preference for story-driven games. We used a user-centered design process that included youth cocreation through an interactive activity where participants developed compelling storylines informed by their lived experiences. Thematic analysis of these narratives informed the development of 3 choose-your-own-adventure style digital storylines focused on HIV disclosure, stigma, and health literacy created in collaboration with a professional game studio. The intervention package additionally includes the provision of PST (Table 1), task-shifted from clinical psychiatrists to HIV adherence counselors who are an integral part of the existing infrastructure of the study site’s HIV clinic (and many other HIV clinics in this environment). This component of the intervention is further described in the Provider Training section.

**Table 1:** Problem-solving therapy (PST) session outline.

Session	Patients receive PST alone	Patients receive PST and Change My Story
Session 1	Psychoeducation and introduction to PST.	Psychoeducation and introduction to PST and Change My Story game. Selection of narrative for game play based on participants’ life experience or current problem.
Session 2	Administer PST steps and agree on homework tasks.	Administer PST steps supported with experience from game play: discuss similarities, differences, and outcome expectations. Agree on homework tasks and narrative for gameplay.
Sessions 3-5	Administer PST steps and review homework tasks.	Administer PST steps supported with experience from gameplay. Review homework tasks and reflect on experience with gameplay.
Session 6	Summarize and reinforce lessons learnt. Assess with PHQ-9^a^ to determine further treatment plan.	Summarize and reinforce lessons learnt and parallels from the game narrative. Assess with PHQ-9 to determine further treatment plan.

^a^PHQ-9: 9-item Patient Health Questionnaire.

### Choice of Comparators

Arm 1 will receive the outlined PST intervention delivered by adherence counselors based in the HIV clinic. Arm 2 will receive the outlined PST intervention integrated with the Change My Story game. Both arms will offer phone-based PST for selected sessions to improve accessibility. We anticipate that both arms will show improvement in mental health outcomes assessed using the 9-item Patient Health Questionnaire (PHQ-9; secondary outcome). However, we hypothesize that integrating PST with Change My Story will improve implementation outcomes (primary outcomes) including satisfaction, feasibility, acceptability, and appropriateness (from the perspective of study participants). It is through these mechanisms that we believe individuals in arm 2 will have greater engagement (primary outcome) in the intervention and improved preliminary effectiveness on both participants’ PHQ-9 score and HIV viral load compared to those in arm 1.

### Provider Training

Prior to recruitment, HIV adherence counselors and physicians will be trained in depression identification, documentation, and management, including support and referral protocols for patients with suicidal ideation or severe depression. HIV counselors will additionally receive training on the delivery of the manualized PST intervention package developed by Gureje et al [15]. Study physicians will receive further training on the appropriate risk assessment, treatment, and referral requirements for high-risk patients using a format that has been successfully used to train nonspecialists and to improve knowledge, attitudes toward mental illness, and competency in diagnosing and treating depression [15,41,42]. To effectively integrate Change My Story with PST delivery, the counselors in this arm will participate in a workshop where they have an opportunity to play the game, review key narratives and themes, identify how narrative themes may relate to important PST principles that can be highlighted in treatment sessions, and role-play PST sessions with and without Change My Story.

### Eligibility Criteria

Trained study staff will approach consecutive attendees at the Infectious Disease Institute while they are waiting to see their health care providers or during youth club sessions. These staff will screen interested participants for eligibility by administering validated screening tools for depression (PHQ-9) as well as for bipolar and psychotic disorder (adapted from the World Mental Health Composite International Diagnostic Interview [WMH CIDI]) in a private area of the waiting room [43-46]. Patients will meet the inclusion criteria if they (1) are HIV seropositive, (2) are 16 to 24 years of age, (3) possess a self-reported proficiency in reading and understanding English, and (4) have depressive symptoms (defined as PHQ-9≥9) [45]. Patients will be excluded if they are pregnant or nursing, have been on antiretroviral therapy (ART) for 6 months, have been out of care for ≥6 months, or have a history of or positive assessment for bipolar or psychotic disorder (based on adapted WMH CIDI) [46]. Individuals who meet eligibility criteria will be invited to provide written informed consent. Consenting individuals with a PHQ-9 score between 9 and 17 will be randomized. Further, individuals with a score of 12-17 will be referred to a clinic physician to ensure that no further specialized psychological assessment is indicated. Those with a PHQ-9 score ≥18 or who are assessed to have serious suicidal risk (ie, indicate thoughts of suicidality) will be immediately referred to a psychiatrist to be evaluated for safety and treatment needs; in these 2 scenarios, if individuals are cleared by the clinic physician or psychiatrist, they may be reconsidered for study eligibility and randomized.

### Randomization

Participants with a PHQ-9 score ≥9 will be randomized 1:1 in a parallel group design by the study statistician to study arm 1, PST alone, or study arm 2, PST with Change My Story. Randomization will be stratified on 2 key baseline variables: age category (16-19 vs 20-24 years) and HIV mode of transmission (perinatal vs nonperinatal). Younger people living with HIV in LMICs may have more limited access to technology, which may impact the feasibility of the mHealth platform [47]. Perinatally and nonperinatally infected young people living with HIV often differ in psychosocial contexts and stigma experiences, which may influence both their engagement with the intervention and mental health outcomes [48]. The random allocation sequence will be generated centrally by the study statistician and implemented using sequentially numbered, opaque, sealed envelopes to ensure allocation concealment until the point of assignment.

### Blinding

Due to the nature of the intervention, it will not be possible to blind participants or implementers.

### Procedures (Both Study Arms)

#### PST (Study Arms 1 and 2)

**Figure 1:**
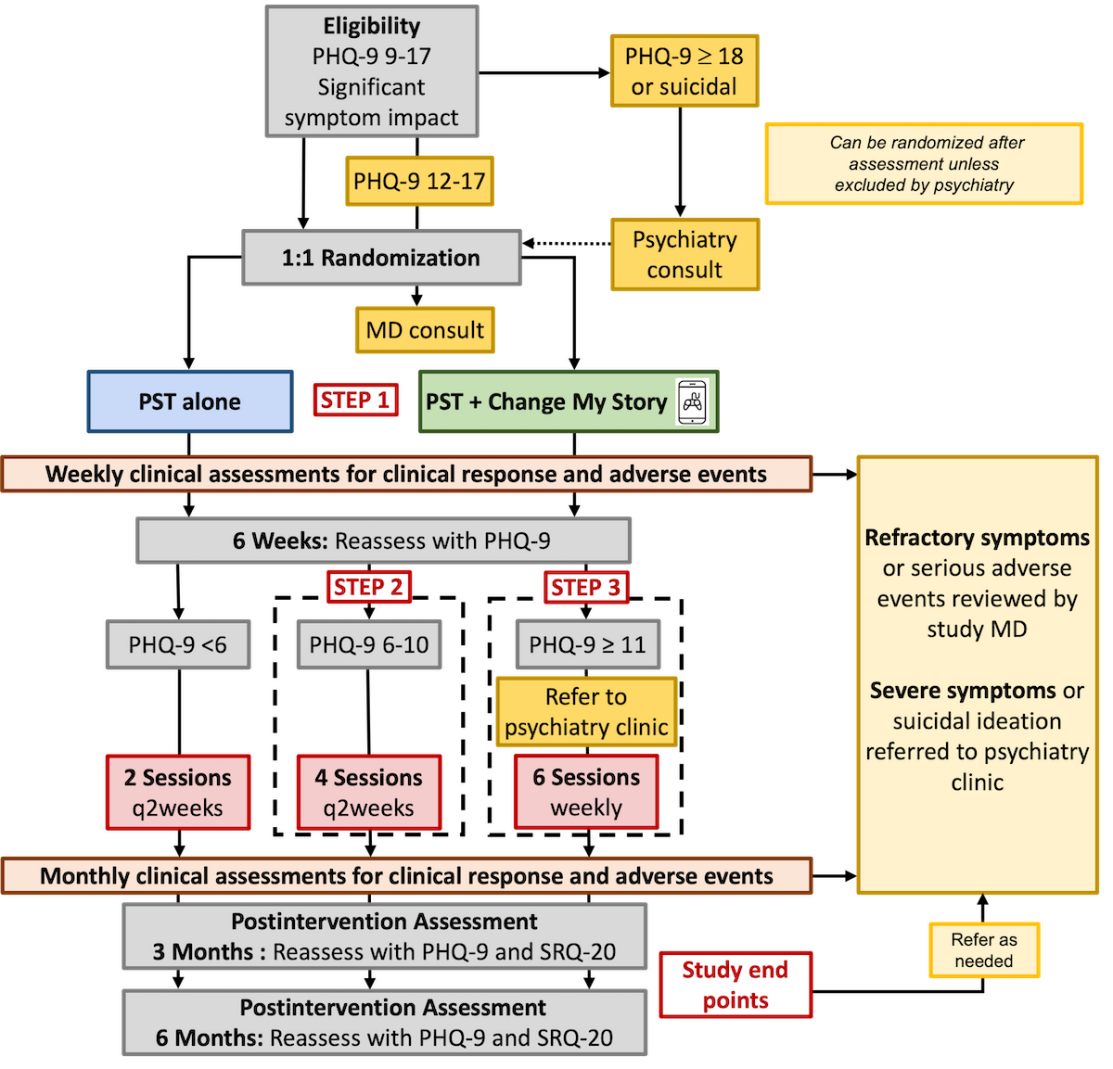
Study schema. MD: medical doctor; PHQ-9: 9-item Patient Health Questionnaire; PST: problem-solving therapy; SRQ-20: Self-Reporting Questionnaire-20.

All participants will receive PST delivered using a stepped-care approach. Figure 1 outlines this study schema. PST appointments will be prescheduled and last approximately 30 to 45 minutes. The first, second, and sixth intervention sessions will be delivered face-to-face in the clinic to facilitate introductions and familiarity with the PST steps. The other sessions will be administered via mobile phone or in person (based on participant preference). The first PST session will include a discussion that will facilitate the generation of a problem list, which will be the focus of subsequent sessions. The first 6 weeks, referred to as “step 1,” involve weekly PST sessions (Table 1).

Subsequent care will be determined based on the participant’s PHQ-9 score at the week 6 evaluation. Participants with a PHQ-9 score <6 will be considered to be in remission and will receive 2 additional PST sessions delivered every 2 weeks. Participants with a PHQ-9 score of 6-10 will be considered to have mild to moderate depressive symptoms and will receive 4 additional sessions delivered every 2 weeks; this is titled “step 2.” Participants with a PHQ-9 score ≥11 will be considered to have moderate to severe depressive symptoms and will receive an additional 6 sessions of weekly PST; this is titled “step 3.” Participants whose PHQ-9 score is >17 or who express suicidality will be referred for evaluation at the psychiatry clinic. All participants who do not achieve remission (PHQ-9 score <6) after completion of the additional 6 weekly therapy sessions will be required to have a referral to either the general medical doctor or psychiatrist at the Infectious Disease Institute to determine the need for pharmacologic treatment or additional intervention based on local clinical guidelines (Figure 1) [14,41,49].

#### PST With Change My Story (Study Arm 2)

At enrollment, participants randomized to study arm 2 will download the game onto their phones, receive information about the game structure or layout, and receive recommendations on how to play the game. Participants will be asked to play through at least 1 of the 3 narratives each week, within 48 hours of the PST session. Participants will be encouraged to set a weekly reminder alarm for their gameplay session. Participants will receive the PST intervention as described in the PST (Study Arms 1 and 2) Section. Counselors will be encouraged to use the participant’s game experience as a tool to help introduce and guide the patient through difficult discussions to facilitate the PST sessions. As such, during the first PST session, the counselor and client together will choose a narrative story that might resonate with the participant’s background and concerns. In subsequent sessions, the patient and counselor may reflect on the patient’s experience with the game, discuss the patient’s thoughts and feelings about the character’s narrative path, and use this discussion to help identify similarities or differences between the character’s and participant’s life challenges. As they identify and work through the participant’s problem list and different problem-solving strategies, the counselor may encourage the participant to play a particular character again and explore a different narrative path to illustrate key features of problem orientation; this may allow the patient to see how characters achieve desired outcomes, reframe their own outcome expectations, and promote self-efficacy in problem-solving. We believe that patients in this study arm will be more engaged in the therapeutic process, have a more satisfying intervention experience, and thus be more likely to complete the recommended PST sessions.

#### Ongoing Evaluation for Suicidality or Serious Adverse Events

During each PST session, the counselor will ask the participant structured questions to identify those at risk of suicide. Participants who indicate suicidality or ongoing refractory disease at reassessment points will require immediate consultation by the Infectious Disease Institute psychiatry clinic. The psychiatry team will determine whether the patient requires initiation or escalation of pharmacotherapy or other intervention [41,50].

### Outcome Assessment

The study will use a type II hybrid design to assess both implementation and effectiveness outcomes across study arms. Outcome assessors will receive training on administering data collection instruments.

The primary outcomes for this study are implementation outcomes (engagement, satisfaction, feasibility, acceptability, and fidelity) and will be compared across participants in PST alone to PST with Change My Story arms*.* Engagement in the intervention is defined as the percentage of recommended PST sessions attended. Satisfaction with the intervention is based on an adapted version of the Client Satisfaction Questionnaire (CSQ-8) [51]. Feasibility and acceptability as well as facilitators and barriers to achieving implementation and clinical outcomes—from the patient’s perspective—will be assessed via focus group discussion (FGD) as well as via Weiner’s Feasibility of Intervention Measure (FIM) and Acceptability of Intervention Measure (AIM) [52]. For feasibility, programmatic data, including the proportion of consented patients enrolled, proportion of game play sessions interrupted by technical problems, proportion of users requiring a mobile phone (at baseline), a replacement phone (due to loss or theft), or data needs, will also be assessed. For acceptability, the number of minutes and number of days of total game play per month, the percentage of recommended game play minutes completed, and the proportion of narratives completed will also be assessed from aggregated game play metrics. We will assess fidelity by determining whether core components of the PST intervention are implemented as planned and as detailed in the intervention manual via direct observation of a random subset of 15% of PST sessions (2 and 5) rated on several dimensions according to a scale that has successfully been used by the study team in Nigeria [53].

Secondary outcomes are clinical outcomes (remission of depression or psychological distress, ART adherence, and viral suppression). Specifically, remission will be defined by a reduction in PHQ-9 from baseline to follow-up (PHQ-9 score <6) [43,44]. Functioning across 6 domains will be measured via the WHO Disability Assessment Schedule [54]. HIV outcomes, including ART adherence and viral suppression, will be, respectively, measured via pharmacy refills and laboratory values (HIV RNA <200 copies per milliliter) at baseline and 3 to 6 months after intervention completion. Laboratories will be drawn for this assessment if participants do not have a routine clinical viral load obtained within this window.

### Quantitative Data Collection

#### Participant Quantitative Data Collection

Trained study staff will administer a battery of widely validated instruments to participants at baseline, 3 months, and 6 months. The battery includes tools to assess depression (PHQ-9 and WMH CIDI), psychological distress (WHO Self-Reporting Questionnaire-20), HIV stigma (Berger’s HIV Stigma Scale), social support (Social Provisions Scale-24), food insecurity (Household Food Insecurity Access Scale), functioning in individuals with psychiatric disorder (WHO Disability Assessment Schedule), and substance-related risks in adolescents (Car, Relax, Alone, Forget, Family/Friends, and Trouble Questionnaire screening test; Table 2) [43,44,46,54-58].

**Table 2:** Participant data collection.

Outcome and measure				Baseline		3 months		6 months
**Baseline covariates**								
	**Demographics**							
		Questionnaire	✓					
	**Psychological distress**							
		SRQ-20^a^ [44]	✓		✓		✓	
	**Depression**							
		PHQ-9^b^ [43]	✓		✓		✓	
		WMH CIDI^c^ [46]	✓					
	**HIV stigma**							
		Berger Stigma Scale [55]	✓					
	**Social support**							
		Social Provision Scale [56]	✓					
	**Food insecurity**							
		Household Food Insecurity Access Scale [57]	✓					
	**Disability**							
		WHO^d^ Disability Assessment Schedule [54]	✓		✓		✓	
	**Substance abuse**							
		CRAFFT^e^ Scale [58]	✓					
	**Clinical data**							
		CD4+^f^ count, HIV viral load, ART^g^ adherence	✓		✓		✓	
**Primary outcomes**								
	**Engagement**							
		Percentage of sessions attended			✓			
	**Satisfaction**							
		CSQ-8^h^ [51], FGD^i^			✓			
	**Feasibility, acceptability, appropriateness**							
		Weiner’s FIM^j^, AIM^k^, IAM^l^ surveys [52]; enrollment rate; mobile phone or data needs; game play metrics; FGD			✓			
**Secondary outcomes**								
	**ART adherence**							
		Percentage of pharmacy refill	✓		✓		✓	
	**HIV viral load**				✓		✓	

^a^SRQ-20: Self-Reporting Questionnaire-20.

^b^PHQ-9: 9-item Patient Health Questionnaire.

^c^WMH CIDI: World Mental Health Composite International Diagnostic Interview.

^d^WHO: World Health Organization.

^e^CRAFFT: Car, Relax, Alone, Forget, Family/Friends, and Trouble Questionnaire.

^f^CD4+: cluster of differentiation 4+ T-lymphocyte cell.

^g^ART: antiretroviral therapy.

^h^CSQ-8: Client Satisfaction Questionnaire.

^i^FGD: focus group discussion.

^j^FIM: Feasibility of Intervention Measure.

^k^AIM: Acceptability of Intervention Measure.

^l^IAM: Intervention Appropriateness Measure.

#### Implementor Quantitative Data Collection

We will administer pre- and posttest knowledge assessments after initial training (focusing on screening, identification, and treatment for depression and components of PST). We will also conduct a baseline readiness assessment, including Rohrbach’s readiness tool and the Organizational Readiness for Implementing Change tool, and assess pre-implementation feasibility, acceptability, and appropriateness from health facility leaders (n=2), the HIV clinic providers (n=4), and HIV counselors (n=4-5) [59,60]. At 3 months, we will administer an assessment of implementation outcomes to this group, which will include Weiner’s Intervention Appropriateness Measure, FIM, and AIM [52]. In addition, the scalability of the intervention will be assessed using the Intervention Scalability Assessment Tool (Table 3) [61].

**Table 3:** Implementer data collection.

Implementation outcome	Measure	Baseline	3 months	6 months
Workshop training assessments	Pre- or posttest, evaluation form	✓		
Readiness	Rohrbach and ORIC^a^ tools [58,59], FGD^b^	✓	✓	
Patient engagement	FGD		✓	
Feasibility	Weiner’s FIM^c^ [51], FGD	✓	✓	
Acceptability	Weiner’s AIM^d^ [51], FGD	✓	✓	
Appropriateness	Weiner’s IAM^e^ [51], FGD	✓	✓	
Fidelity	Direct observation	Ongoing	Ongoing	Ongoing
Scalability	Intervention Scalability Assessment Tool [60]		✓	

^a^ORIC: Organizational Readiness for Implementing Change.

^b^FGD: focus group discussion.

^c^FIM: Feasibility of Intervention Measure.

^d^AIM: Acceptability of Intervention Measure.

^e^IAM: Intervention Appropriateness Measure.

### Qualitative Data Collection

#### Participant Qualitative Data Collection

FGDs will be conducted with study participants (n=20) at the study end. Our FGD guide was developed using the CFIR framework and focused on determinants of key implementation outcomes (feasibility, acceptability, satisfaction, and engagement) of our patient-facing strategies and environment [36,62]. We will examine outer setting factors, such as IT infrastructure, to enable digital game use, community-level stigma, and sociocultural norms influencing mental health and HIV care. Inner setting constructs will explore participants’ perceptions of the clinic environment and its support for mental health services. Characteristics of individuals will include participants’ knowledge, beliefs, and self-efficacy related to using the digital game and engaging in PST. Intervention characteristics will be examined through perceptions of the game’s relevance, quality, and usability and the coherence between the game content and PST sessions. Finally, implementation process factors, such as clarity of instructions, support received, and game-clinic integration, will be assessed to understand what facilitated or hindered engagement. The full guide can be found in Multimedia Appendix 1. These qualitative insights will inform the contextual appropriateness and potential scalability of the intervention.

#### Implementer Qualitative Data Collection

We will conduct 2 FGDs with health facility leaders (n=2), HIV clinic providers (n=4), and HIV counselors (n=4-5), once during pre-implementation to assess knowledge and attitudes about mental health, PST, and readiness for the intervention, and again at the study end to assess implementation outcomes and their determinants. As with the participants, the CFIR will guide our assessment of implementation outcome determinants, appropriateness and feasibility of implementing this strategy in Nigeria (outer setting), the clinic’s culture and attitudes toward mental health screening and treatment, internal capacity to support mHealth-based interventions, impacts of the intervention on other services (inner setting), knowledge and beliefs about the intervention, confidence in implementor’s abilities to administer the intervention and integrate the game-based strategy into the intervention (provider characteristics), effectiveness of provider training, effectiveness of integration of the strategy into clinic workflow, facilitators and barriers to intervention fidelity (process of implementation), and complexity and quality of the intervention and implementation strategies (intervention characteristics), challenges, as well as contextual factors influencing acceptability and satisfaction with the intervention [36,62]. The full guide can be found in Multimedia Appendix 2.

### Sample Size and Power Considerations

With a total sample size of 80 participants (40 per study arm), the study will have approximately 80% power to detect a difference between the intervention and control groups equivalent to 0.635 SDs in the outcome measure using a 2-sided *t* test with a significance level of .05.

#### Engagement in the Intervention

Using prior data from a study on PST for depression and common mental disorders among adults in Zimbabwe in which the SD for the number of sessions attended was 18.5%, we anticipate having 80% power to detect a difference of 11.7% in the mean proportion of sessions attended between the study arms [24]. If the SD in our study population is as low as 10% or as high as 25%, then we will have approximately 80% power to detect mean differences in the proportion of sessions attended of 6.3% and 15.8%, respectively. If up to 20% (16/80) of participants are lost to follow-up (LTFU), then we anticipate having 80% power to detect a difference of 13.2% in the mean proportion of sessions attended between the study arms.

#### Satisfaction With the Intervention

While there are no preliminary data in similar populations, in diverse populations receiving PST, the SD of the CSQ-8 (scores range from 8 to 32) was 4.78-4.95 [63,64]. Assuming SDs of 4.5, 4.75, and 5.0 in the CSQ-8 scores and no LTFU, we anticipate having 80% power to detect differences of 2.9, 3.0, and 3.2, respectively, in the mean scores between study arms. With 20% (16/80) LTFU, these estimated detectable differences are 3.2, 3.4, and 3.6, respectively. While we will not be powered to detect differences in our secondary clinical outcomes in this pilot RCT, we believe the value of this intervention approach may not necessarily come from changing PST efficacy but from improving adoption and engagement for young people living with HIV with the intervention, thus improving its overall effectiveness or so-called mosaic effectiveness [65].

#### Retention

To promote retention and minimize LTFU, we will use established clinic strategies including phone reminders for appointments and targeted outreach by clinic or study staff for missed visits. To reduce participation burden, only 3 PST sessions will be required to be in person, others can be delivered via mobile phone (per participant preference), and participants will receive travel reimbursement and refreshments for the face-to-face sessions and outcome assessments. Participants who elect to discontinue from the study will be asked to complete an exit visit, which will include the assessments outlined in Table 2.

### Data Analysis

#### Overview

Primary outcomes include (1) engagement in the intervention defined as the percentage of recommended PST sessions attended, (2) satisfaction with the intervention using the CSQ-8, (3) feasibility, and (4) acceptability (from the patient perspective; Table 2). These outcomes will be collected throughout the course of the study through engagement with the app and participation in PST sessions. Exceptions include the CSQ-8, FIM, and AIM, which will be administered at 3 months, and the fidelity checklist, which will be applied to 2 separate sessions during the study.

Our secondary outcomes include remission of depression, level of mental distress, disability, ART adherence, and HIV viral suppression (Table 2). These outcomes will be assessed at 3 and 6 months (viral suppression will be assessed once between 3 and 6 months).

#### Quantitative Data Analysis

Engagement and satisfaction scores (primary outcomes) and ART adherence (secondary outcome) will be compared between study arms using a Wilcoxon rank sum test to avoid making distributional assumptions. Additional secondary outcomes (remission of depression and psychological distress and viral suppression) will also be compared using chi-square tests. We will compare primary and secondary outcomes between study arms using semiparametric cumulative probability models. Because of the relatively small sample size, baseline covariates will be included using a propensity score to account for any covariate imbalance between the treatment arms. Missing covariate data, if any, will be multiply imputed with 20 imputation replications using chained equations. Primary analyses will be intention-to-treat analyses; we will also perform secondary, as-treated analyses. Persons who are out of care will be assumed to have been nonadherent over the period for which they were out of care, and intervention engagement over these gaps will be assigned the value 0. Secondary analyses will exclude person-time when individuals are not in care. Descriptive statistics will be used to summarize the ordinal responses obtained from FIM and AIM surveys in addition to programmatic and game log data measuring other features of feasibility and acceptability. We will assess other factors associated with the study primary and secondary outcomes, in addition to potential confounders and mediators of the relationship between the intervention and outcome including baseline sex, cluster of differentiation 4+ T-lymphocyte cell count, age, marital status, psychological distress, HIV stigma, social support, food insecurity, quality of life, and substance abuse [43,44,55,57,66-70].

#### Qualitative Data Analysis

After gaining consent, we will record and transcribe all interviews. Transcripts will be read by 2 individuals to ensure that they are complete and adequately address the research questions as well as to develop an initial codebook. The first 5 interviews will be pulled to assess the identification of expected deductive codes identified from the CFIR framework and relevant literature [36]. Unexpected codes will be generated and defined for future coding. Coding will be conducted, as interviews are completed to ensure we reach data saturation. We will assess intercoder agreement. Coding and analysis will be done using MAXQDA (version 12; VERBI), with a directed content analysis approach informed by the 5 CFIR domains [36].

#### Data Integration

We will use a convergent parallel mixed methods design to integrate qualitative and quantitative data [71]. After initial independent analysis of both patient and implementer datasets as described earlier, the multidisciplinary team will examine the quantitative and qualitative data analyses concurrently to identify areas where the findings converged, diverged, or added insight to one another to inform assessments of our hypothesis that participants who receive PST integrated with Change My Story will have improved implementation and effectiveness outcomes compared to those receiving PST alone. We will additionally assess emergent themes that can further inform determinants of study outcomes. Data integration discussions, particularly addressing any concerns with divergent results, will include both the broader study team and youth advisors to ensure contextual validity and the creation of additional data collection plans if deemed necessary.

### Quality Control Mechanisms

The quality of the study outputs will be ensured by using validated data collection tools. In addition to the creation and implementation of operations manuals, data collectors and supervisors will be trained on all data collection tools and procedures. In total, 1 research assistant will manage the mobile app technology to ensure proper functioning, 2 Nigerian-based research assistants will coordinate the overall research, and 1 US-based research assistant and US-based program manager will provide continuous supervision throughout the entire follow-up. All case report form data will be stored on the University of Ibadan secure network drives and captured onto a secure, password-protected REDCap (Research Electronic Data Capture; Vanderbilt University) database. The encrypted REDCap data servers will be hosted by the University of Ibadan. Electronic mobile technology use data will be stored on a back-end storage system at the University of Ibadan that meets Health Normative Standards Framework guidelines, which call for restricted access and the separation of patient demographics and health data.

Recognizing that loss of privacy is a serious issue, especially for an mHealth-grounded intervention for youths, measures will be put in place to protect identifiable data. First, all investigators and research personnel will receive training on the protection of human participants. Second, identifiable information will only be made available to a few specific research personnel who will need access to the data purely for study purposes. All paper records will be stored in secure, locked cabinets only accessible to study personnel. Third, the mHealth app was developed with security as the top priority. The security features include early deidentification of data through the assignment of participant ID numbers, storage of data in an encrypted format, secure HTTPS-based data transmission between the mHealth app and a password-protected, Health Insurance Portability and Accountability Act (HIPAA)–compliant cloud-based storage system, robust user-level authentication to access the app, and automatic log-out after a period of inactivity. To minimize risk to confidentiality and privacy, the app will not include any explicit indications to its purpose as an HIV intervention. From an outside perspective, Change My Story will appear like other general apps, without obvious associations to mental health or HIV-related services. Fourth, participants will be counseled on the low risk of data breach with these safeguards, but also on how to further minimize data breach when phones are shared. Participants will additionally be taught to use the app and participate in remote PST sessions in ways that protect their privacy. These safeguards will be present in the demonstration session, introducing the participants to the app. Nonetheless, these risks will be explicitly discussed with participants at the time of enrollment and consent for study participation. Given the limited scale of the study, the research team will maintain direct oversight of all procedures and data collection; no additional monitoring processes are anticipated.

### Ethical Considerations

This study involves human participants and was reviewed and approved by the Mass General Brigham Institutional Review Board (IRB) in the United States (Protocol #: 2024P000857) and the University of Ibadan IRB in Nigeria (Protocol #: UI/EC/24/0484). Written informed consent will be obtained from all participants prior to enrollment. Individuals aged 16 years and older as well as emancipated youths can consent for research in compliance with Nigerian regulations and IRB requirements. The consent process will include a clear explanation of the study’s purpose, procedures, potential risks and benefits, voluntary nature of participation, and the right to withdraw at any time without penalty. All participants will receive due incentives and refreshments for in-person study visits (including outcome assessments) to account for their time and transportation needs. Participants randomized to the integrated PST and Change My Story arm will receive monthly mobile carrier bundles during the intervention period to ensure data access required for study activities. These reimbursements are intended solely to offset costs incurred as a result of participation and are not considered coercive. No performance-based or outcome-dependent incentives will be provided. All participant data will be deidentified through assignment of unique study identification numbers prior to storage or analysis. The Change My Story mobile app was developed with security as a priority, using encrypted data storage, secure HTTPS-based data transmission, robust user authentication, and automatic log-out.

### Dissemination

The findings from this proposal will be shared as abstracts at international HIV conferences and submitted for publication in peer-reviewed journals. At the conclusion of the trial, we will consolidate findings and prepare a presentation oriented to an audience of trial participants and other young people living with HIV at the Infectious Disease Institute.

### Data Monitoring Committee

A Data Safety and Monitoring Board has been formed to safeguard the interests of participants. The Data Safety and Monitoring Board is an independent advisory group that will provide recommendations. The members have expertise in clinical psychiatry, HIV clinical trials, adolescent medicine, and biostatistics.

### Patient and Public Involvement Plan

End users and key clinical and public health partners in the field of HIV have been integrally involved in the development of the Change My Story game. Throughout the data collection and follow-up period, the end users along with clinical and public health partners will provide key perspectives on outlined implementation outcomes in addition to how they can be overcome to maximize patient benefit.

## Results

This study is funded by the US National Institutes of Health (funding commenced on March 8, 2024), and IRB approval was received on April 15, 2024. Participant recruitment and data collection began in June 2025. Through this study, we will assess our hypothesis that participants who receive PST integrated with Change My Story will have improved implementation and effectiveness outcomes compared to those receiving PST alone. Thus far, we have screened 103 youths and enrolled 23 of a planned 80 trial participants. Among enrolled participants, 15 (65%) were male, 1 (4%) had a PHQ-9 score ≥17, and 6 (26%) had suicidal thoughts underscoring the need for routine mental health screening and intervention in this population. We anticipate recruitment to be completed by January 2026 and follow-up to be completed by June 2026.

## Discussion

Nigeria and other LMICs are facing overlapping epidemics of HIV and mental disorders that have gone largely unaddressed despite their well-documented interrelationship [72,73]. Depression is common among young people living with HIV, a finding underscored by our early enrollment data, and is strongly associated with ART nonadherence, LTFU, and virologic failure [4-7]. Although CBT interventions such as PST are effective for depression in both high-income and LMIC settings [11-16,22,24], their scalability is limited by a shortage of mental health specialists [17-19]. Task-shifting PST to trained lay providers can improve access, but sustained engagement in multisession therapy remains challenging for young people living with HIV due to mobility constraints, stigma, and discomfort discussing mental health in clinic settings [5,6,23].

This pilot trial addresses these challenges by integrating PST with Change My Story, a culturally tailored, narrative-based serious game designed with youth input to provide a low-stigma, youth-friendly entry point to care [25-27]. Serious games have demonstrated promise for health behavior change [30-35], and narrative formats can enhance identification, observational learning, and self-efficacy [31,32,37,38]. However, prior HIV-related gaming interventions have largely focused on prevention rather than mental health, and few have been embedded within evidence-based psychotherapy for young people living with HIV in LMICs. By combining a stepped-care, task-shifted PST model with a behaviorally grounded digital game [37-39], this study targets 2 major implementation gaps: limited scalability of specialist-delivered care and poor retention in psychotherapy among youths [22,24].

While our approach addresses important barriers to engagement, it does not directly address social determinants of health (such as poverty, housing instability, food insecurity, gender-based violence, and structural stigma) that also shape adherence and mental health outcomes [5,6,55,57,66]. mHealth platforms offer a low-friction strategy to deliver psychosocial support, which may reduce some care access barriers (when delivered remotely) and equip young people living with HIV with coping skills to better manage and navigate these persistent stressors [11,12,33]. Our mixed methods design, guided by the CFIR, will allow us to identify unaddressed barriers, including those reflecting social and structural determinants, and inform adaptations for future iterations of the intervention [36].

The primary limitation of this study is its modest sample size as a pilot trial, which will limit statistical power to detect changes in secondary clinical outcomes and may reduce generalizability. Recruitment from a single, well-resourced HIV clinic with established youth-friendly services may also limit applicability to other settings with fewer resources. To address this, we plan to conduct supplementary FGDs with young people living with HIV and stakeholders from other regions in Nigeria to assess transferability. Despite these limitations, this trial will yield critical early evidence on the feasibility, acceptability, and preliminary effectiveness of an mHealth-enhanced, task-shifted psychological intervention for young people living with HIV, filling a major evidence gap in the literature. Findings from the trial will advance the literature on integrating digital innovations into mental health care for HIV and—if effective—may inform scalable strategies to close the persistent gap between innovation and impact in global HIV and mental health care.

## Appendix

Multimedia Appendix 1 Participant focus group discussion guide.

Multimedia Appendix 2 Implementer focus group discussion guide.

## Abbreviations

AIM: Acceptability of Intervention Measure
ART: antiretroviral therapy
CBT: cognitive behavioral therapy
CFIR: Consolidated Framework for Implementation Research
CSQ-8: Client Satisfaction Questionnaire
FGD: focus group discussion
FIM: Feasibility of Intervention Measure
HIPAA: Health Insurance Portability and Accountability Act
IRB: institutional review board
LMIC: low- and middle-income country
LTFU: lost to follow-up
mHealth: mobile health
PHQ-9: 9-item Patient Health Questionnaire
PST: problem-solving therapy
RCT: randomized controlled trial
REDCap: Research Electronic Data Capture
WHO: World Health Organization
WMH CIDI: World Mental Health Composite International Diagnostic Interview

## Data Availability

The datasets that will be generated during this trial are available from the corresponding author on reasonable request.
