# Supplementary material for: A Mobile Game Intervention for Young Persons Living With HIV and Depression in Nigeria: Protocol for a Pilot Randomized Controlled Trial
Source: JMIR Res Protoc. 2025 Dec 3;14:e74199. doi: 10.2196/74199 (PMC12712569; doi:10.2196/74199)
Supplement: Multimedia Appendix 1 [file resprot_v14i1e74199_app1.docx]

**Participant Follow-Up FGD Guide**

**How did you find the overall experience of participating in the Change My Story intervention?**

Probe: playing the game, interaction with counselors, staff, etc

**In what ways did the intervention impact your mental health (depression, anxiety)? What about your *thoughts* on mental health?**

Probe: Which parts of the intervention impacted your mental health or *thoughts on* mental health? (eg talks with counsellor, playing game, strategies for problem solving from counselor or game characters)

**Where there things about the intervention that you found particularly helpful? If so, what, why?**

Probe: sessions with the counselors, doctors, playing the game, discussing game experience with counselors, etc

**What were some things that made it easy for you to participate in the Change My Story intervention?**

Probe: Game (if in game arm), Counselor style/relationship, frequency of meetings, type of meetings (phone vs. in-person), clinic characteristics/culture, transportation, phone/data/IT needs

**What were some things that made it difficult for you to participate in the Change My Story intervention? How would the study team address those challenges?**

Probe: Difficult conversations, Counselor style/relationship, frequency of meetings, type of meetings (phone vs. in-person), clinic characteristics/culture, transportation, phone/data/IT needs

**What concerns did you have about your privacy when participating in this intervention? How can we address those concerns?**

Probe: office space, staff interaction/reaction, game play, phone calls/contacts, etc

**Would you recommend the Change My Story intervention to other young people with HIV and challenges with their mood? Why or why not?**

***For Individuals Randomized to Play the Change My Story Game***

**Can you share your thoughts on the digital Change My Story game?**

Probe: Did you feel that the game adequately addressed the specific issues faced by young people living with HIV? Why or why not? How did the game impact your understanding of the importance of mental health care in managing HIV treatment outcomes? How well did the game and your sessions with the counselor relate/work together? What issues were not addressed by the game?

**What did you like about the Change My Story Game?**

**Probe**: Which specific storylines or characters (length, appearance/user interface, comic format, plot) etc.

**What did you dislike about Change My Story Game?**

**Probe**: Which specific storylines or characters (length, appearance/user interface, comic format, plot) etc.

**What challenges did you have using the game? How could we better address/have addressed these challenges?**

**Would you recommend the Change My Story game to other young people with HIV and challenges with their mood? Why or why not?**
