# Supplementary material for: A Mobile Game Intervention for Young Persons Living With HIV and Depression in Nigeria: Protocol for a Pilot Randomized Controlled Trial
Source: JMIR Res Protoc. 2025 Dec 3;14:e74199. doi: 10.2196/74199 (PMC12712569; doi:10.2196/74199)
Supplement: Multimedia Appendix 2 [file resprot_v14i1e74199_app2.docx]

**Change My Story Implementer Study-End FGD Guide**

**What was the general level of receptivity in your organization to implementing the intervention? Why?**

Probes: What about your personal receptivity? Other influential stakeholders (clinical leadership, institutional leadership, supervisors)?

**Do you think the intervention was effective for improving mental health for young people with HIV in your setting? Why or Why not?**

**How well do you think the intervention met the needs of the individuals served by your organization?**

Probes: In what ways did the intervention meet their needs?  In what ways did the intervention not meet their needs (probe on needs influencing mental health not addressed by intervention including social determinants of health)? How do you think patients responded to the intervention?

**How did playing and integrating the Change My Story digital game influence PST delivery?**

Probes: Improved engagement, made it more complex, did not influence PST, etc.?

**How much did participants like or dislike the intervention?**

Probes: Can you describe specific examples of this?

**What barriers did YPWH face to participating in the intervention?**

Probes: Consider tech savviness, IT infrastructure, transportation, PST sessions over the phone, availability for frequent PST sessions, etc, mental health stigma, relationship with counselor

**How does the intervention compare to other similar existing programs in your setting?**

Probes: What advantages or disadvantages does Change My Story have to these other programs? To what extent would implementing the intervention provide an advantage for your organization compared to other organizations in your area?

**Was the intervention implemented according to the protocol?**

Probes: [If Yes] Can you describe this? [If No] Why not? Probe on facilitators/barriers in the outer setting (IT infrastructure, stigma), inner setting (clinic logistics including competing needs), implementation process, and participant needs/barriers.

**What kinds of changes or alterations were required to the intervention so it would work effectively in your setting?**

Probes: Address complexity, additional resource (people, money), etc? How easy or challenging was it to integrate the intervention into your workflow? What could have made the intervention stronger?

**How confident are you that you successfully implemented the intervention?**

Probes: How prepared were you to conduct the intervention? What would have made you feel more prepared?

**What would be required to sustain this intervention in your setting?**
